# The Decimating Army: Hemophagocytic Lymphohistiocytosis as the Initial Presentation of Adult-Onset Still’s Disease

**DOI:** 10.7759/cureus.43875

**Published:** 2023-08-21

**Authors:** Walter Y Agyeman, Susan Waitimu, Kofi D Seffah, Saint-Martin Allihien, Saheed Soleye

**Affiliations:** 1 Internal Medicine, Piedmont Athens Regional Medical Center, Athens, USA

**Keywords:** adult-onset still’s disease, reactive hemophagocytic syndrome, macrophage activation syndrome, immune activation, hemophagocytosis, hemophagocytic lymphohistiocytosis (hlh)

## Abstract

Hemophagocytic lymphohistiocytosis (HLH) is a rare and often missed presentation in the hospital setting. It is a hyperinflammatory syndrome of immune activation and dysregulation characterized by fever, cytopenias, elevated serum ferritin levels, and hepatosplenomegaly. It has a multifactorial etiology occurring primarily secondary to infection, malignancy, immune checkpoint inhibitors, or autoimmune disease. HLH that occurs in autoimmune diseases such as adult-onset Still’s disease and systemic lupus erythematosus is referred to as macrophage activation syndrome. However, it may rarely be a primary disorder from a genetic defect. The pathophysiology of HLH is poorly understood and often results in multiorgan dysfunction. We present an older adult male patient with several hospital stays due to his symptoms, who presented with lethargy, low-grade fever, cough, dyspnea, and recurrent pleural effusions. He had bicytopenia, elevated ferritin, and hypertriglyceridemia. The diagnosis of HLH may be delayed, especially in older adult patients with an insidious course, and requires a high index of suspicion.

## Introduction

Hemophagocytic lymphohistiocytosis (HLH) is an infrequent and often missed presentation in the hospital setting. HLH is a hyperinflammatory syndrome caused by excessive cytokine release, triggered by genetic or acquired overactivation of macrophages, T lymphocytes, and natural killer (NK) cells [1]. It is a syndrome characterized by immune activation and dysregulation leading to clinical manifestations such as fever, cytopenias, elevated serum ferritin levels, and hepatosplenomegaly. It is classified into primary/hereditary (pHLH) and secondary/acquired (sHLH). Its multifactorial etiology is primarily secondary to infection, malignancy, immune checkpoint inhibitors, or autoimmune disease. However, it may rarely be a primary disorder from a genetic defect. Secondary HLH can be initiated by various insults that activate the immune system, such as infections, autoimmune diseases, and tumors. Epstein-Barr virus (EBV) is a common pathogenic factor of HLH, accounting for about 70% of infection-associated HLH, and the most common cause of tumor-associated HLH is non-Hodgkin’s lymphoma [2]. The pathophysiology of HLH is poorly understood and often results in multiorgan dysfunction. Management is based on high-dose steroid therapy and etoposide with or without intrathecal chemotherapy. Hematopoietic stem cell transplants (SCT) are done in selected cases.

## Case presentation

**Figure 1:**
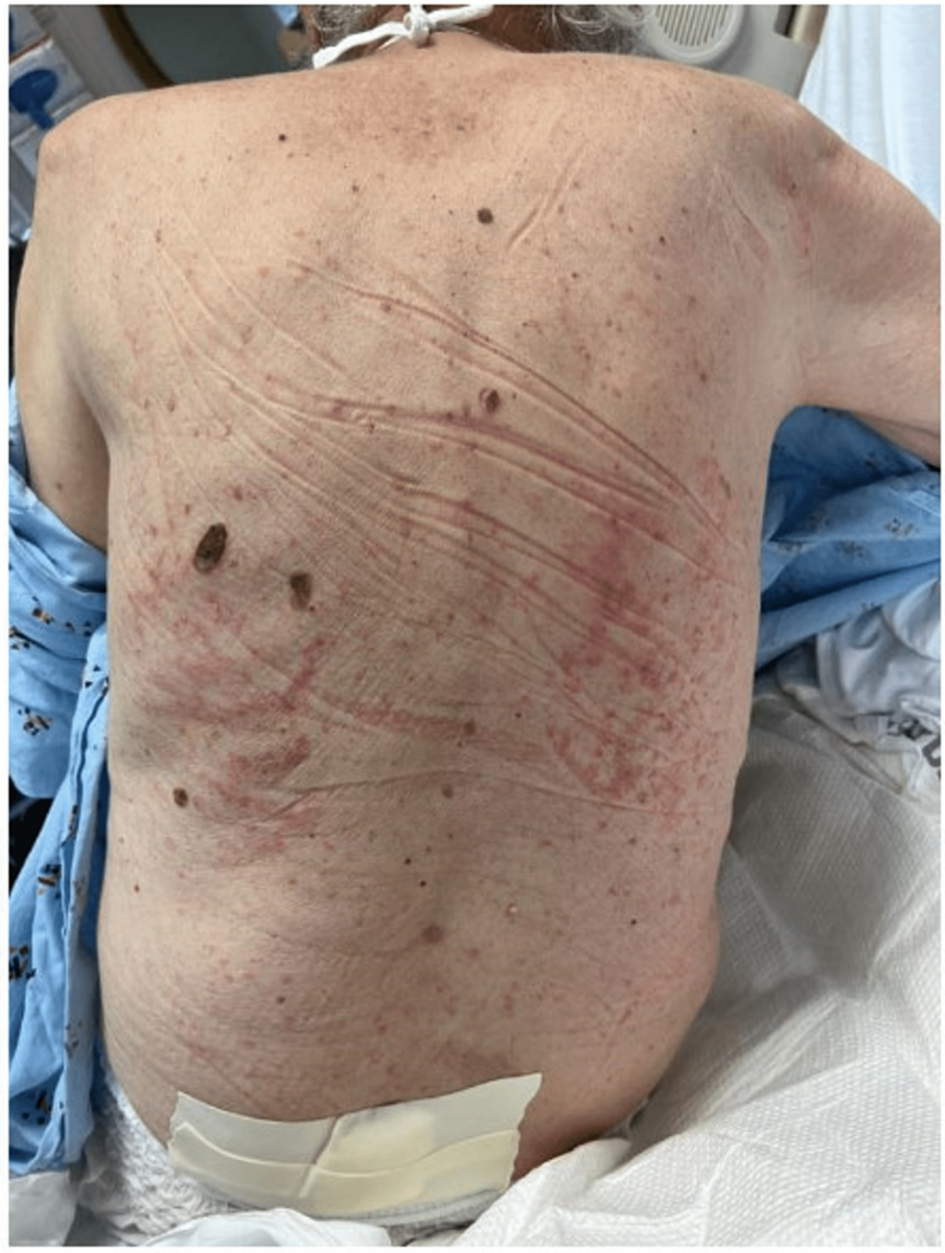
Salmon pink-colored rash

A 79-year-old Caucasian male presented to the hospital with dyspnea, recurrent low-grade fever, and lethargy. He had been hospitalized three weeks prior with similar symptoms and managed for a parapneumonic effusion with a prolonged three-week course of antibiotics. He presented the day after completing the antibiotic course with persistent symptoms and unintentional weight loss of about 30 pounds over two months. There was dyspnea with exertion, but no associated cough, sputum production, wheezing, hemoptysis, or pleuritic chest pain. His medical history was significant for arthritis, multiple benign small bowel, and maxillary sinus tumors, atrial fibrillation on Xarelto, chronic obstructive pulmonary disease, benign prostatic hyperplasia, hypertension, and a nasopharyngeal mass biopsy showing chronic inflammation without malignancy. His surgical history was notable for a polypectomy from the small intestine. On initial presentation, he was hypotensive, tachypneic, and tachycardic with irregular rhythm with significant findings of blood pressure of 92/58 mmHg, heart rate of 112 beats/minute, respiratory rate of 22 breaths/minute, and temperature of 96.3°F. He was chronically ill-looking and had a firm, non-tender left submental enlarged lymph node. He also had absent breath sounds at the left lower lung zone with stony dull percussion. He had a salmon pink-colored rash (Figure 1).

**Figure 2:**
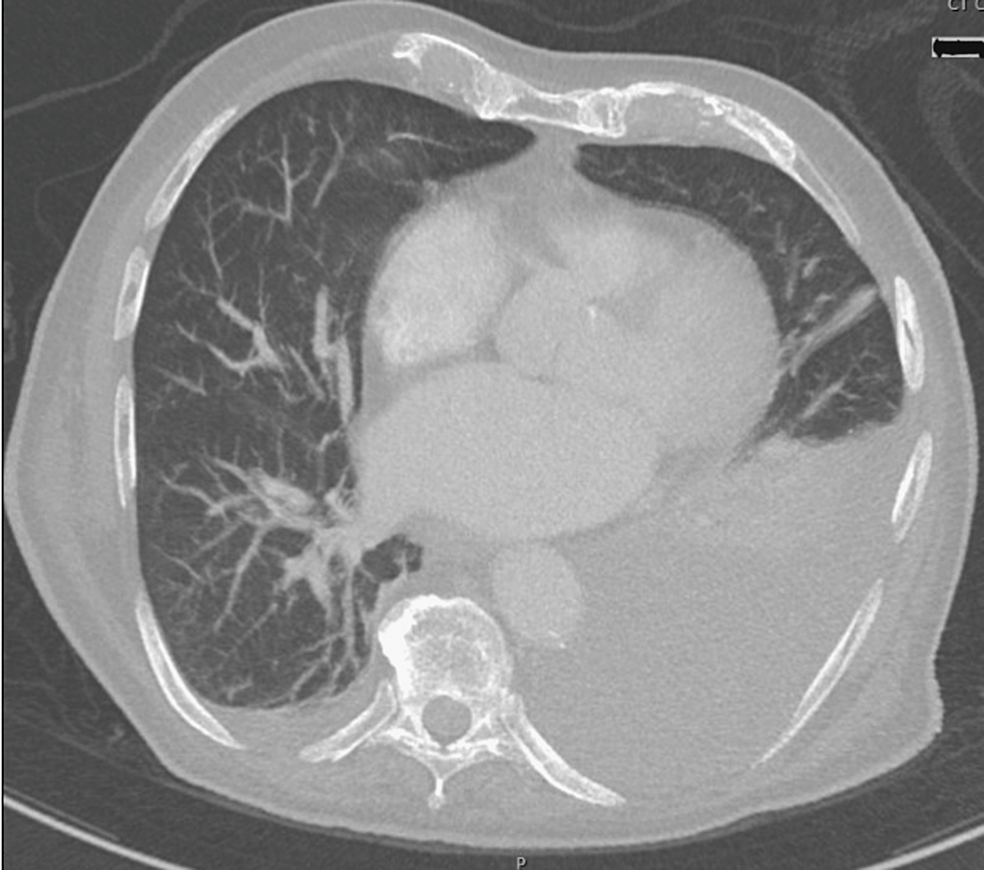
CT of the chest without contrast showing a left pleural effusion CT: computed tomography

His laboratory tests were significant for a white blood cell (WBC) count of 2,100 × 10^3^/µL, hemoglobin of 12.5 g/dL, and platelet of 49,000 × 10^3^/µL (Table 1). He had serum lactate of 3.2 mmol/L, an international normalized ratio (INR) of 2.44, creatinine of 1.1 mg/dL, and procalcitonin of 0.28 ng/mL. Chest imaging showed an increased left pleural effusion compared to prior imaging three weeks ago (Figure 2).

**Table 1:** Hematologic laboratory data

	Presenting laboratory result	Nadir on admission	Reference unit
Hemoglobin	12.5	5.5	g/dL
White blood cell count	2,100	1,100	×10^3^/µL
Platelets	49,000	27,000	×10^3^/µL
Fibrinogen	57	57	mg/dL

Initial thoracocentesis two weeks prior revealed an exudative pattern with negative cytology. A repeat thoracocentesis this admission again showed an exudative pattern with cytology showing atypical T lymphocytes and an elevated adenosine deaminase level of 65 U/L. Flow cytometry of the pleural fluid was negative for a neoplastic B-cell population. The infectious workup was remarkable for positive Epstein-Barr viral IgG. Acid-fast bacilli culture, *Aspergillus* antigen, histoplasma, and respiratory cultures were negative.

He was monitored off antibiotics and evaluated by the oncology service for pancytopenia. A bone marrow biopsy done three weeks previously was unremarkable. He was also evaluated by the infectious disease service, who had a very low suspicion of an infectious cause of his exudative pleural effusion. He rapidly deteriorated two days after admission with worsening hypoxemia and the development of atrial fibrillation with a rapid ventricular response. He was transferred to the intensive care unit (ICU) with a severe systemic inflammatory response. He continued deteriorating with worsening hypotension and persistent fever with a maximum temperature of 103.8°F. He also had a progressively worsening WBC count of 1,600 × 10^3^/µL, hematocrit of 22%, and platelets of 38,000 × 10^3^/µL. He had bilateral pleural effusions L>R with associated compressive atelectasis with acute hypoxemic respiratory failure. He continued to spike daily intermittent fevers. He developed multiorgan dysfunction with acute metabolic encephalopathy, profound metabolic acidosis with elevated lactic acid, bandemia, positive EBV serology, hyperferritinemia, hypertriglyceridemia, markedly elevated transaminitis, and disseminated intravascular coagulation, raising suspicion for hemophagocytic lymphohistiocytosis secondary to EBV. He met three major (fever > 39°C for >1 week, typical salmon pink-colored rash, and arthralgias of >2 weeks) and three minor (submental lymph node enlargement, negative antinuclear antibody (ANA), rheumatoid factor, and elevated aspartate aminotransferase (AST)) Yamaguchi criteria, which were diagnostic of adult-onset Still’s disease with 96.2% sensitivity and 92.1 specificity [3]. Still’s disease has been associated with HLH. His workup revealed ferritin of >7,500 ng/mL, triglycerides of 271 mg/dL, fibrinogen of 57 mg/dL, and AST of 108 U/L. Other significant laboratory parameters are noted in Table 2. His H-score was 226, meeting a 96%-98% probability of HLH. The HLH-94 protocol consisting of etoposide and dexamethasone was initiated. Unfortunately, his condition deteriorated, and his family opted for comfort measures. He succumbed three days later.

**Table 2:** Significant laboratory data INR: international normalized ratio, CRP: C-reactive protein, AST: aspartate aminotransferase, ALT: alanine aminotransferase, LDH: lactate dehydrogenase, RBC: red blood cell, pH: acidity/power of hydrogen, ANCA: antineutrophil cytoplasmic antibodies, MPO: myeloperoxidase, GBM: glomerular basement membrane, ANA: antinuclear antibodies, AFB: acid-fast bacilli, TB: tuberculosis, EIA: enzyme-linked immunoassay, IgM: immunoglobulin M, IgG: immunoglobulin G, DNA: deoxyribonucleic acid, PCR: polymerase chain reaction, EBV: Epstein-Barr virus, VCA: viral capsid antigen, Ep-CAM: epithelial cell adhesion molecule, TTF-1: thyroid transcription factor-1, CDX2: caudal-type homeobox 2, CD20: cluster of differentiate 20, PAX5: paired box 5, CD30: cluster of differentiation 30, ALK1: activin A receptor-like type 1, CD5: cluster of differentiation 5

Laboratory parameter	Result	Reference range
Reticulocyte count	1%	0.5%-2%
INR	2.44	<1.1
CRP	2.1 mg/dL	<0.3 mg/dL
Vitamin B12	454 pg/mL	211-950 pg/mL
D-dimer	4,300 ng/mL	<500 ng/mL
Fibrinogen	153 mg/dL	200-393 mg/dL
Serum ferritin	>7,500 ng/mL	24-336 ng/mL
Total bilirubin	1.6 mg/dL	0.2-1.4 mg/dL
Direct bilirubin	0.96 mg/dL	0-0.3 mg/dL
Triglycerides	271 mg/dL	<150 mg/dL
AST	1,603 U/L	12-50 U/L
ALT	914 U/L	7-52 U/L
Creatinine	1.37 mg/dL	0.5-1.2 mg/dL
Lactate	7.5 mmol/L	<2 mmol/L
Serum LDH	1,200 U/L	140-280 U/L
Pleural fluid color and appearance	Yellow and hazy	Straw-colored
Pleural fluid nucleated cells/mm^3^	45/mm^3^	<1,000 /mm^3^
Pleural fluid RBC/mm^3^	4,773/mm^3^	<100/ mm^3^
Total protein pleural fluid	3.2 g/dL	<4.1 g/dL
Pleural fluid pH	7.4	7-7.5
Pleural fluid glucose	81 mg/dL	70-110 mg/dL
Pleural fluid LDH	863 U/L	70-200 U/L
Pleural fluid adenosine deaminase	63.5 U/L	<9.2 U/L
ANCA screen with MPO PR-3 reflex titer	Negative	Negative
Anti-GBM antibodies	Negative	Negative
ANA screen	Negative	Negative
Anticyclic citrullinated peptide antibodies	<16 units	<20 units
Blood culture	No growth after five days	No growth
Pleural fluid culture	Negative	Negative
Sputum for AFB growth	Negative	Negative
Sputum gram stain	Negative	Negative
Sputum AFB stain	Negative	Negative
T spot TB test	Negative	Negative
Quantiferon-TB	Indeterminant	Negative
Aspergillus antigen index	0.17 units	<0.5 units
Aspergillus antigen EIA	Not detected	Not detected
Histoplasma antibodies	1:16	<1:8
Histoplasma urine antigen	<0.2 ng/mL	<0.2 ng/mL
Fungitell 1-2 B-D-glucan	98 pg/mL	<60 pg/mL
Parvovirus IgM and IgG antibodies	Negative	Negative
Hepatitis panel	Negative	Negative
Cytomegalovirus antibody IgM	<30 AU/mL	<30 AU/mL
Cytomegalovirus DNA quantitative PCR	54 IU/mL	Not detected
Epstein-Barr virus DNA PCR	1483 copies/mL	Not detected
EBV VCA IgM	<36 U/mL	<36 U/mL
EBV VCA IgG	406 U/mL	<18 U/mL
EBV EBNA IgG	>600	Not detected
Pleural fluid cytology comment	Large, atypical cells on the smears and cell block section show multinucleation, large nuclei, prominent nucleoli, and positivity for CD3 immunostain. Scattered macrophages are seen. Mesothelial cells are not conspicuous. Flow cytometry did not disclose an abnormal B-cell population.	Not detected
Flow cytometry	The large, atypical cells are positive for CD3 immunostain. Pancytokeratin, Ep-CAM, TTF-1, cytokeratin 7, cytokeratin 20, CDX2, CD20, PAX5, CD30, ALK1, and CD5 immunostains are negative in the large, atypical cells. Mucicarmine histochemical stain is negative.	Not detected

## Discussion

Hemophagocytic lymphohistiocytosis (HLH) was first described in 1939 by Scott and Robb-Smith [4]. HLH is a rare disorder characterized by a dysregulated immune response with activation of macrophages, cytotoxic T-cells, and natural killer (NK) cells leading to excessive cytokine release and immune-mediated multiple organ injury [5]. It is often a fatal disease, especially in the adult population. The diagnosis of HLH can be challenging and requires a high index of suspicion. Definitive diagnosis is usually delayed due to its atypical and varied presentation in the initial stages. It shares similarities with other hyperinflammatory states, such as sepsis or multiorgan dysfunction. Pathological evaluation of a bone marrow sample with the identification of hemophagocytic histiocytes clinches the diagnosis. However, marrow evaluation for hemophagocytic histiocytes usually correlates poorly with disease probability [6].

HLH is classified into primary/hereditary (pHLH) and secondary/acquired (sHLH). Primary HLH encompasses a spectrum of genetic disorders with HLH as the predominant manifestation. These familial HLH disorders include pathological changes in perforin 1 (*PRF1*), unc-13 homolog D (*UNC13D*), syntaxin 11 (*STX11*), and syntaxin-binding protein 2 (*STXBP2*) (familial HLH2-HLH5) [7]. These genetic diseases are optimally treated with allogeneic hematopoietic cell transplantation (HCT). Several other genetic diseases, including certain pigmentary disorders (Ras-related protein Rab-27A (*RAB27A*), lysosomal trafficking regulator (*LYST*), and adaptor-related protein complex 3 subunit beta 1 (*AP3B1*) genes), X-linked lymphoproliferative diseases (SH2 domain containing 1A (*SH2D1A*) and X-linked inhibitor of apoptosis (*XIAP*) genes), specific cell division cycle 42 (*CDC42*) mutations, and activating mutations in NLR family CARD domain containing 4 (*NLRC4*) are also associated with HLH [7]. Secondary HLH may be initiated by various insults that trigger the immune system. Secondary HLH can occur following infection, malignancy, immune checkpoint inhibitors, or autoimmune disease. Most information on the diagnosis and treatment of HLH is derived from the pediatric literature. This presents several challenges when applying diagnostic and management protocols to adult cases. The HLH-2004 diagnostic criteria were developed in a pediatric population with primary HLH. Although most physicians use these criteria to diagnose adults, it has not been validated in adults with secondary HLH [1]. A diagnosis of HLH is met in the HLH-2004 study if patients had five out of eight clinical criteria. The most recent criteria include fever, splenomegaly, cytopenias, hypertriglyceridemia and/or hypofibrinogenemia, hemophagocytosis, decreased natural killer (NK) cell function, elevated ferritin, and elevated soluble IL-2 receptor levels [7].

Patients with HLH may present with clinical features similar to sepsis or multiple organ dysfunction syndrome [8]. This presents a significant challenge to the accurate and early diagnosis of HLH in adults. Our initial suspicion for HLH in the index patient was raised due to persistent fever with a maximum temperature (Tmax) of 104°F and pancytopenia. Our patient met four out of eight HLH-2004 criteria, specifically pancytopenia, hypertriglyceridemia, ferritin > 500 ng/mL, and fever > 104°F. He had significantly elevated ferritin of >7,500 ng/mL, the highest cutoff for our laboratory. HLH-2004 criteria ≥ 5 predicted HLH with a sensitivity of 91% and a specificity of 93% [2]. An increase of the ferritin cutoff value to 3,000 μg/L and a fever cutoff value of 38.2°C increases diagnostic sensitivity and specificity to 97.5% and 96.1%, respectively [1]. A serum ferritin cutoff value of 2,000 µg/L can be used in isolation to diagnose HLH with a sensitivity of 70% and a specificity of 68% [9]. This higher cutoff value of ferritin may help distinguish HLH from sepsis and other hyperinflammatory diseases.

H-score is a readily available probability tool validated in adults with reactive or secondary HLH. An H-score ≥ 200 has been demonstrated to have excellent diagnostic accuracy for HLH in overall and critically ill populations [1]. This optimal cutoff of ≥200 performed comparably to the HLH-2004 criteria, although an H-score of ≥169 predicted HLH with better sensitivity (96%) but reduced specificity (71%) [2]. Our patient’s H-score was 226. Epstein-Barr virus IgG antibodies and viral DNA were highly positive, and we believe that this was another immune trigger for our patient, as this is the cause of 70% of infection-associated HLH [2]. He had a chronic Epstein-Barr infection in the setting of Still’s disease. Our patient had a bone marrow biopsy done six weeks before that did not show hemophagocytosis. Still, at that time, cytopenias were very mild and likely before clinically significant marrow changes had occurred. Depending on the time it is done, marrow evaluation may miss the presence of hemophagocytic histiocytes and not correlate with disease probability [6]. A repeat marrow assessment was not necessary as his HLH pretest probability was high enough to make the diagnosis and justify treatment. He had HLH-associated multiorgan dysfunction. He had a high likelihood of HLH with an H-score associated with a 93%-96% probability of HLH.

The lack of data to guide evidence-based management is a common challenge in treating adults with HLH. Treatment algorithms are frequently based on pediatric protocols based on data extrapolated from children, such as HLH-94 and HLH-2004 protocols. This often results in overtreatment and unnecessary toxicity in adults. These older adult patients with chronic comorbidities are more susceptible to end-organ damage caused by the cytokine storm in HLH and the HLH-94 chemotherapy. It is, therefore, prudent to tailor the length and dosing of the HLH-94 treatment plan by considering associated comorbidities and initiating triggers in adults. A definitive cure may be achieved in adults with primary HLH using allogeneic hematopoietic SCT (alloHSCT), as evidenced by dramatically improved outcomes in children with this therapy [8].

Treatment according to the HLH-94 protocol, without alloHSCT, appears beneficial in secondary HLH [10]. The HLH-94 algorithm includes eight weeks of initial therapy to achieve clinical remission, followed by continuation therapy. The initial therapy consists of etoposide (150 mg/m^2^ twice weekly for two weeks and then weekly) and dexamethasone (initially 10 mg/m^2^ for two weeks followed by 5 mg/m^2^ for two weeks, 2.5 mg/m^2^ for two weeks, 1.25 mg/m^2^ for one week, and one week of tapering) [10]. The continuation therapy from week 9 is composed of a regimen of dexamethasone pulses 10 mg/m^2^ for three days every second week and etoposide infusions 150 mg/m^2^ every alternating second week in combination with daily oral cyclosporine A, aiming at trough levels of 200 μg/L [10]. Intrathecal methotrexate is administered at a maximum of four doses only if progressive neurological symptoms exist. The index patient received pulsed dose steroids and was then started on the HLH-94 protocol as etoposide 150 mg/m^2^ twice weekly for two weeks and intravenous dexamethasone 10 mg/m^2^ for the first two weeks. The dose of etoposide was reduced, considering his decreased renal function. Intrathecal methotrexate is given when there is evidence of central nervous system disease. This was withheld from our patient due to his coagulopathy. It is recommended to provide prophylactic antimycotic treatment and *Pneumocystis jirovecii* prophylaxis during the initial chemotherapy phase [10]. Usually, response to the chemotherapy regimen can be tracked by following the fever curve and improvements in involved end-organ dysfunction. His clinical condition continued to deteriorate while on the HLH-94 protocol. His poor baseline performance status, advanced age, comorbidities, and severe HLH disease-related multiorgan injury were too significant, making treatment challenging. Given his advanced age and competing all-cause mortality rate, even the best response to HLH treatment was unlikely to change his long-term prognosis. In this case, continued aggressive therapy was not meaningful to the patient’s family. The patient’s family subsequently opted for a conservative approach with supportive treatment.

## Conclusions

HLH is a rare and insidious disease that poses a diagnostic challenge, often leading to delayed diagnosis as it mimics many hyperinflammatory disorders. Timely diagnosis using the HLH-2004 criteria and H-score is crucial due to its associated high morbidity and mortality. High ferritin scores also serve as a prognostic marker, with high values signifying a higher mortality rate. A higher cutoff value of ferritin may help in distinguishing HLH from sepsis. The index patient presented with features of adult-onset Still’s disease and had highly positive antibodies to the Epstein-Barr virus with a significant viral load. Epstein-Barr virus is a known infectious trigger for HLH. Given this disease’s high morbidity and mortality, evidence-based clinical guidelines for adult HLH management should be developed.
